# Quantifying single-cell responses to irradiation in 3D

**DOI:** 10.3389/fbioe.2026.1735308

**Published:** 2026-03-25

**Authors:** Joshua François, Alina Simerzin, Ashwini Jambhekar, Galit Lahav

**Affiliations:** Department of Systems Biology, Blavantik Institute, Harvard Medical School, Boston, MA, United States

**Keywords:** 3D, imaging, matrigel assay, microscopy, p21 (CDKN1A), spheroids

## Abstract

**Introduction:**

Understanding how cells respond to internal and external inputs requires investigating cells within three-dimensional (3D) environments, which better mimic physiological conditions. Compared to two-dimensional (2D) systems, 3D cultures more accurately simulate tissue architecture, including cell-cell and cell-extracellular matrix interactions, as well as gradients of oxygen and nutrients. Despite these advantages, quantifying signaling dynamics in 3D remains difficult due to limitations in imaging depth, phototoxicity, and computational analysis.

**Methods:**

We developed experimental and computational tools for tracking individual cells’ responses in 3D. We focused on the response of human breast cancer cells to irradiation using a cell line that expresses a fluorescent reporter for the cell cycle regulator p21, which is activated by the tumor suppressor p53 after irradiation. We embedded individual cells and multicellular spheroids in a dual-Matrigel assay and used light sheet fluorescence microscopy (LSFM) to obtain high-resolution images at several time points post-irradiation. We then developed computational pipelines to obtain detailed reconstructions and quantitative analyses of p21 dynamics.

**Results:**

Individual dispersed cells exhibited a gradual, monotonic increase in the fraction of p21-positive cells, with the majority of cells becoming positive 24 h after irradiation. When applied to spheroids, the same system captured a transient decrease in the fraction of p21-positive cells post-irradiation, followed by a delayed pronounced rise only at 24 h. In addition, while the fraction of p21-positive cells increased in both systems, p21 intensity within induced cells remained relatively constant. This behavior is consistent with studies in 2D cultures showing that irradiation induces p53 oscillations, with each p53 pulse regulating the probability, rather than the magnitude, of p21 transcription. Notably, spatial mapping of annotated nuclei showed no dependence between p21 levels and radial cell position within spheroids. Comparisons between 2D, 3D single-cell, and spheroid data indicate that while the overall extent of p21 activation is similar across systems, the kinetics differ, with spheroids exhibiting slower induction.

**Discussion:**

The differences in features such as p21 induction kinetics observed in spheroids compared to 2D and 3D single-cell cultures post-irradiation likely reflect p21 signaling specific to cells in 3D configurations with cell–extracellular matrix constraints. Overall, the platform developed in this study provides a powerful framework to dissect heterogeneous signaling dynamics in physiologically relevant 3D contexts and can be extended to assess the effects of drug treatments on other complex multicellular structures.

## 1 Introduction

Three-dimensional (3D) experimental models have emerged as essential tools for understanding how cells respond to external inputs across a wide range of biological processes, from development to cancer progression and responses to drug treatments. Such models vary in scale, ranging from single cells in three-dimensional scaffolds, to spheroids and organoids, to multiple cell types in bioprinted tissues (Xie et al., 2024; Moysidou et al., 2021). A major advantage of these models is their ability to recapitulate the complex 3D interactions found in physiological environments, such as cell-cell interactions and cell-substrate interactions with extracellular proteins. These interactions can significantly influence cellular responses to external stimuli in ways that differ from those observed in traditional two-dimensional (2D) cell cultures (Bhuker et al., 2025; Zhang et al., 2025; Imamura et al., 2015). For example, therapies targeting breast cancers that overexpress Human Epidermal Growth Factor Receptor 2 (HER2) are less effective when HER2-overexpressing cancer cells are grown in 3D versus 2D environments (Weigelt et al., 2010; Breslin and O’Driscoll, 2016). Notably, the sensitivity of these cancer cells to HER2-targeted drugs increases following inhibition of β1 integrin, a key cell-ECM receptor, highlighting the critical role of 3D cell-matrix interactions in modulating drug responses (Weigelt et al., 2010). The 3D architecture of multicellular structures can also create oxygen and nutrient gradients, leading to hypoxic conditions and subsequent induction of hypoxia-inducible factor 1 (HIF-1), which promotes chemoresistance in breast cancer cells (Doublier et al., 2012). These examples highlight the importance of studying biological signaling responses within 3D environments and utilizing 3D models to more accurately capture physiologically relevant behaviors.

Numerous experimental models now enable the investigation of 3D signaling responses, including approaches that achieve single-cell or time-resolved readouts (e.g., hypoxia-driven tumorigenesis and early neurogenesis) (Wang et al., 2025a; Wang et al., 2025b). However, implementing assays that robustly capture signaling dynamics at single-cell resolution in 3D still faces important practical and technical challenges. Scaffold-based approaches, for example, involve embedding individual cells or spheroids in 3D hydrogels, resulting in their random dispersion, which can make locating cells during imaging labor-intensive and increases the likelihood of out-of-plane light scattering, further complicating image acquisition (Richardson and Lichtman, 2015; Frantz et al., 2022). While multi-hydrogel layer assays may address this by confining samples to a narrow range of z-planes, phototoxicity from conventional 3D imaging modalities, such as confocal microscopy, can perturb signaling dynamics, particularly in systems sensitive to DNA damage (Tallapragada et al., 2021; Chow et al., 2024). Furthermore, many studies rely on immunofluorescence staining of fixed 3D samples, a process which, even with advanced optical clearing methods, risks compromising spheroid integrity through dehydration and delipidation steps. This may result in the disruption of spatial relationships between cells and preclude the ability to track dynamic cellular responses to external perturbations (Nürnberg et al., 2020).

Computational challenges have also limited the broader adoption of 3D models and systems. Most computational tools for analyzing signaling responses were originally designed for 2D systems, and their application to 3D imaging datasets can introduce biases depending on which regions are selected for imaging and analysis. In addition, achieving high spatial resolution in 3D microscopy is technically challenging, and the sheer volume of data generated by high-resolution imaging can be difficult to manage and process. As a result, measurements of signaling responses in 3D models often rely on averaging signal intensity across entire spheroids, which masks cellular heterogeneity. These limitations underscore the need for advanced computational tools capable of robustly analyzing 3D datasets, particularly at the single-cell level.

In this study, we established experimental and computational platforms for investigating signaling responses in 3D. We focused on the response of cancer cells to irradiation-induced DNA damage given its well-characterized mechanism and central relevance to cancer biology. We developed tools to quantify the behavior of the cell cycle regulator p21, which is transcriptionally induced in response to irradiation by the tumor suppressor protein p53, and plays a key role in halting the growth of cells until DNA is repaired (El-Deiry et al., 1993; Abbas and Dutta, 2009). Previous work on the response of p21 to irradiation in cancer cells grown in 2D showed that p21 transcription behaves as an ON-OFF switch: higher p53 levels primarily influence the probability of transcriptional activation, while only having a minor impact on the magnitude, which rapidly reaches saturation in the ON state (Hafner et al., 2020). Using a dual-Matrigel assay, a live cell reporter for p21 levels, and light-sheet microscopy, we quantified the dynamics of p21 in response to irradiation in 3D-embedded individually dispersed cells and single cells within spheroids. We developed a computational pipeline to reconstruct single cells and cells in multicellular structures in 3D, segment individually dispersed cells embedded in 3D, and measure corresponding p21 signals at the single-cell level. These tools allowed us to extract key features of p21 behavior after irradiation in 3D and compare them to previously published responses in 2D. In the future, similar approaches can be used to investigate the responses of other signaling molecules in 3D environments at the single-cell level.

## 2 Materials and methods

### 2.1 Cell culture

The cell line present in this study (MCF7 – human breast adenocarcinoma), was originally commercially obtained from ATCC [ATCC HTB-22] and engineered to express the fluorescent reporters MCP-YFP and p21-mCherry as previously described (Hafner et al., 2020). Cells were grown at 37 °C and 5% CO_2_ in RPMI-1640 with L-glutamine (RPMI; Corning), supplemented with 10% fetal bovine serum (FBS; Gemini Bio). Cells were grown to 80% confluency and passaged by removing media and washing cells in PBS followed by treatment with 0.05% trypsin for 5 min. Cells were collected in media, pelleted, and resuspended in fresh media for incubation.

### 2.2 Spheroid generation

Spheroids were generated by adding ∼200 MCF7 cells in 200 μL of RPMI supplemented with 10% FBS to each well in ultra-low attachment, low cluster round bottom 96-well plates (Costar). Plates were centrifuged for 1 min at 1,450 rpm before incubation at 37 °C and 5% CO_2_ for 6 days.

### 2.3 Dual-Matrigel assay fabrication for individual cells

Dual-Matrigel assays were constructed by adding 15 μL of thawed phenol-red free Matrigel® matrix basement membrane (Matrigel; Corning, lot no. 17323004) in the center of No. 1.5 thickness 24 × 50 mm glass coverslips (VWR) inside 10-cm petri dishes (Falcon). 1 mL of prewarmed PBS was added to the edge of petri dishes to minimize Matrigel drying and dishes were incubated at 37 °C and 5% CO_2_ for 10 min. MCF7 cultures were washed with prewarmed PBS and trypsinized as described during cell culturing. Approximately 4 × 10^5^ cells were then resuspended in fresh, prewarmed phenol red-free, riboflavin-free RPMI supplemented with 5% FBS before adding 10 μL of the cell suspension on top of the first Matrigel layer. Dishes were incubated at 37 °C and 5% CO_2_ for 30 min to allow cells time to settle on the Matrigel layer. Another 15 μL of Matrigel was then added on top of cells and incubated at 37 °C and 5% CO_2_ for 10 min to form a second Matrigel layer. Finally, 5 mL of phenol red-/riboflavin-free RPMI (Gibco) with 5% FBS was gently added to dish containing the dual-Matrigel system.

### 2.4 Dual-Matrigel assay fabrication for spheroids

For dual-Matrigel assay containing spheroids, the first layer of phenol red-free Matrigel was fabricated as described above. Next, media in wells containing spheroids was partially aspirated. Spheroids were then carefully collected using pipette tips with cut ends to avoid cell dissociation and centrifuged in 1.5 mL Eppendorf tubes at 1,200 rpm for 6 min. Spheroids were gently resuspended in ∼ 10 μL of RPMI with 5% FBS and added on top of the first Matrigel layer. Dishes were incubated at 37 °C and 5% CO_2_ for 30 min to allow spheroids to settle. The second layer of Matrigel was fabricated as described above, followed by the addition of 5 mL of phenol red-/riboflavin-free RPMI with 5% FBS to dish.

### 2.5 Irradiation

Individual MCF7 cells and MCF7-derived spheroids were irradiated in dishes after seeding cells on the bottom Matrigel layers with 10 Gy using an RS-2000 X-Ray irradiator.

### 2.6 Imaging

Volumetric imaging of dual-Matrigel systems was performed using an ASI diSPIM microscope mounted on a Nikon Ti stand, with an Agilent laser launch, Hamamatsu Flash 4.0 v2 camera, and 40× immersion lens objective. Imaging was performed in a single-view configuration. Glass coverslips containing samples were placed in a custom holding chamber, with phenol red-/riboflavin-free RPMI with 5% FBS added. The custom chamber allowed for temperature and CO_2_ control at 37 °C and 5% CO_2_. 150-μm thick volumetric stacks were imaged with 1-μm Z stack spacing between slices, with Z corresponding to the optical axis. Spheroids were qualitatively centered in volume stacks.

### 2.7 Single-cell analysis pipeline

A set of custom MATLAB algorithms were implemented to perform single-cell analysis of p21 levels per unit volume in individually dispersed cells and single-cells within spheroids. The code developed in this study can be made available upon request.

#### 2.7.1 Data conversion

Micro-Manager software was used to export diSPIM imaging time-course volumetric stacks in the form of OME-TIFF files. Custom MATLAB scripts were written to load OME-TIFF files and export binary and TIFF files for each volumetric stack, *V(x, y, z, t)*.

#### 2.7.2 Initial automated segmentations

**FIGURE 1:**
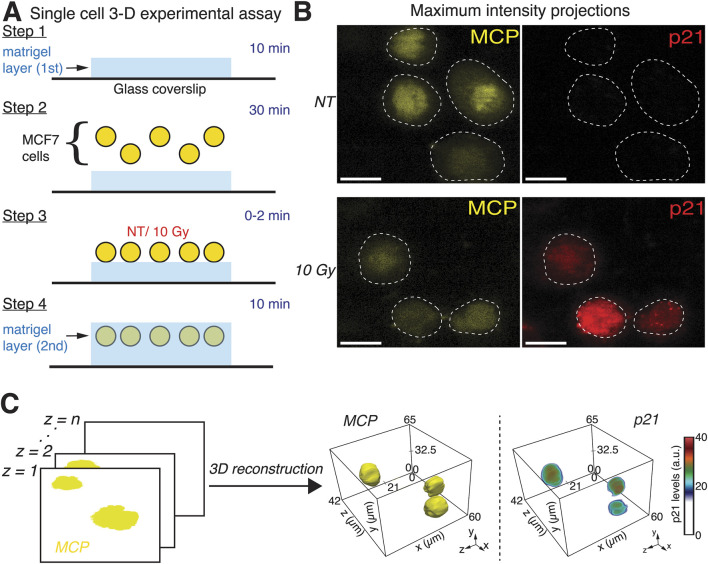
Dual-Matrigel system and 3D single-cell visualization demonstrate irradiation induces p21 in individually dispersed cells. **(A)** Schematic illustration of the experimental assay. Dual-Matrigel layer assay facilitates 3D embedding of human cancer cells in planar configuration. **(B)** Representative maximum intensity projections (MIPs) from volumetric light sheet fluorescence microscopy data of non-irradiated (NT) and irradiated (10 Gy) human breast cancer (MCF7) cells expressing MCP-YFP (nuclear marker) and p21-mCherry fluorescent reporters after 24 h. MIPs illustrate p21 induction in irradiated cells, with heterogeneous levels of expression. Scale bars correspond to 20 μm. **(C)** 3D single-cell reconstruction pipeline. Slices from representative volumetric light sheet data were compiled in MATLAB before being exported as a VTK file for visualization of nuclear channel and the corresponding p21 channel.

An initial pass of coarse segmentations was performed on individually dispersed cells and spheroids to obtain centroids and bounding regions that would be used to subsequently obtain more refined segmentations. First, each Z stack of the MCP-YFP channel, which was used as a nuclear marker, was smoothed by convolving the imaging volumes with a 31 × 31 × 11 pixel box filter. Note that the difference in MCP-YFP fluorescence between control and irradiated cells, as shown in Figure 1B, did not affect our analysis: in all conditions the MCP-YFP signal remained well above background and was sufficient for accurate nuclear segmentation. An initial background calculation, assumed to be 1.5 times the average pixel intensity, was subtracted from the smoothed image. Foreground (pixels >0) and background (pixels <0) were then explicitly defined, and *V(x, y, z, t)* was multiplied by the foreground mask, setting background intensities to 0 in the original volumetric stack. This resulting image was convolved with an averaging filter box size of 15 × 15 × 5 pixels for additional smoothening. Each object with pixels > 0 was then uniquely labeled for volumetric stacks. Objects with pixel sizes smaller than 1 × 10^5^ were removed. Finally, centroids and bounding box coordinates of all remaining objects (cells or spheroids) were stored.

#### 2.7.3 2nd level segmentations

A second pass of segmentations was performed for experiments with individually dispersed cells and spheroids. For volumes with individually dispersed cells, stored bounding box coordinates for segmented cells were used to crop regions around each cell in the YFP channel. A multilevel threshold using Otsu’s method was employed to obtain three thresholds that separated background, pericellular, perinuclear, and nuclear regions. Thresholds for nuclei were defined as pixel values between the threshold corresponding to perinuclear and nuclear regions and were applied in the bounding box region for the cell being segmented. After this second pass of segmentations, all cells were relabeled with unique identification numbers.

For volumes with spheroids, volumetric stacks were smoothed by convolving the stacks with a 31 × 31 × 11 pixel box filter before applying a threshold of 1.5 times the average intensity of the volumetric stack. Noise (debris and loose cells within Matrigel) was removed by counting all identified objects in the imaging volume and removing all objects smaller than 50% of the pixel size of the largest identified object in the volume. A convex hull was then computed to simplify spheroid segmentation boundaries used in later analysis. The complements of masks for segmented individually dispersed cells and spheroids were computed to define volume backgrounds in the mCherry channel. The average intensities of these regions were computed and defined as the final image background value, *B(t)*, for the corresponding timepoint.

#### 2.7.4 3rd level segmentations

A third pass of segmentations was performed for individually dispersed cells by first identifying the maximum YFP intensities within regions defined for each cell in the second-level segmentations in an iterative manner. The locations of these intensities were used as “seed” coordinates for a region-growing algorithm to perform the final segmentation of each cell. A bounding box was created around the newly segmented cells, and previously defined average background intensities from the mCherry channel for the timepoint of interest, *B(t)*, were subtracted from volumes containing the original mCherry data, with the segmentation volumes used as masks. Average mCherry intensities within segmented volumes were then computed to obtain average p21 levels per unit volume in individual cells.

### 2.8 Single-cell annotations for spheroid data

3D annotations for single cells within spheroids were obtained by first loading volumetric TIFF files for individual timepoints of spheroids from their YFP (nuclear) channels into Napari. Nuclei that were reliably distinguishable from other nuclei were manually annotated in all planes, and these annotations were used to assign a specific identifier per nucleus. Volumetric TIFF files containing all annotations for the timepoints of interest were then exported.

### 2.9 Single cell nuclear p21 intensity calculations for spheroid volumetric data

A bounding box was created around each annotated cell within spheroid volumes iteratively. Previously defined mCherry channel background intensities for each timepoint, *B(t)*, were subtracted from volumes containing the original mCherry data, with the annotated volumes used as masks. Average mCherry intensities within segmented volumes were then computed to obtain average p21 levels per unit volume for individual cells within spheroids.

### 2.10 Spatial single-cell analysis for spheroid volumetric data

Cartesian coordinates *(x, y, z)* of each annotated cell within spheroids were computed and spheroids were centered to the middle of imaging volumes for each timepoint. Coordinates were then converted to spherical coordinates to obtain radial distances *ρ* from the center of each spheroid. Normalized radial distances *ρ*_*norm*_ were computed by defining a parametric line from spheroid centroids to the edges of spheroid segmentations, with $0\leq \rho_{norm}\leq 1$ .

### 2.11 Maximum intensity projections

2D maximum intensity projections of light-sheet volumetric data were generated using ImageJ’s Z-Project function, with “Max Intensity” selected for projection type for all 150 volumetric slices acquired.

### 2.12 3D reconstructions

3D reconstructions of individually dispersed cells and spheroids were generated by loading volumetric TIFF files for the YFP and mCherry channels. Custom MATLAB scripts were developed to generate VTK files, which were then imported into ParaView for generating 3D reconstructions.

### 2.13 Statistical analysis

Two-sample *t* tests with a preadjusted P-value of 0.05 were used for all statistical analyses. P-values were Holm-Bonferroni adjusted to account for multiple hypothesis testing.

## 3 Results

### 3.1 Experimental and computational platforms for visualizing and quantifying dispersed single cells’ responses in 3D

To capture the behavior of the cell cycle regulator p21 in 3D-embedded cells in response to irradiation, we designed an experimental system using a modified version of a previously reported multilayer hydrogel assay for high-throughput imaging of encapsulated cells (Cambra et al., 2022). We first fabricated thin layers of Matrigel, a substance rich in ECM proteins, on glass coverslips and seeded individual human breast cancer cells (MCF7) engineered to express the MS2 coat protein (MCP) fused to the yellow fluorescent protein (YFP) as a nuclear marker, and a fluorescent reporter for p21 protein by tagging one allele of p21 with mCherry at its endogenous locus (Figure 1A) (Hafner et al., 2020). MCF7 cells were chosen as our cell model given that they contain two wild-type alleles of the p53 gene, which is a key activator in the DNA damage response pathway (Balcer-Kubiczek et al., 1995). Additionally, the DNA damage response in these cells has been well characterized, enabling a controlled investigation into the effects of local environments on p21 signaling responses. We allowed cells to settle to the bottom of the Matrigel layer for 30 min, irradiated them with 10 Gy, a dose shown to induce DNA damage and a robust p21 response in 2D, and then fabricated the second Matrigel layer (Hafner et al., 2020). Seeding cells between two layers of Matrigel resulted in a relatively planar dispersion of cells, facilitating high-throughput imaging in an environment which, unlike in 2D cultures, cells could engage in 3D cell-ECM interactions.

We imaged unirradiated and irradiated cells using Light Sheet Fluorescence Microscopy (LSFM), which provides high spatial-resolution 3D images, while minimizing phototoxicity and potential DNA damage. Maximum intensity projections of volumetric data showed heterogeneous increases in p21 levels 24 h after irradiation compared to p21 levels in unirradiated cells (Figure 1B). Given that maximum intensity projections may obscure 3D variations in intensities, potentially leading to inaccurate representations of the signal, we developed a custom MATLAB-based pipeline specifically optimized for Matrigel-embedded sample light sheet datasets. The workflow integrates 3D segmentation (background subtraction, adaptive thresholding, and seed/region-growing) and intensity quantification steps. Unlike existing packages, our approach enables batch processing of volumetric OME-TIFF time-course data and direct export to Visualization Toolkit (VTK) format files for 3D visualization in ParaView. 3D reconstructions of cell nuclei (MCP-YFP signal) and their corresponding p21 levels (p21-mCherry signal) 24 h post-irradiation demonstrated that our experimental and computational platform enables the visualization and quantification of p21 expression in single irradiated cells (Figure 1C).

### 3.2 Temporal behavior of p21 in dispersed irradiated single cells in 3D

**FIGURE 2:**
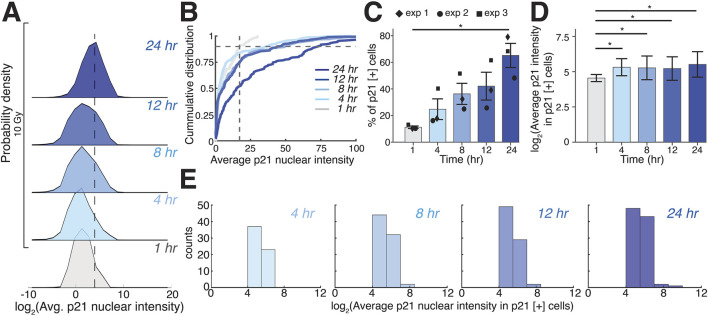
3D single-cell analysis reveals properties of p21 induction in response to irradiation. **(A)** Log2-transformed probability density plots of average nuclear p21 levels in 10 Gy irradiated cells from volumetric light sheet data for 3D-embedded cells at the indicated timepoints post-irradiation. **(B)** Cumulative distributions for p21 levels in cells 1 h post-irradiation used to define a threshold for the top 10th percentile of p21 levels (dashed lines), which was applied across cumulative distributions post-irradiation to calculate the percentage of p21-positive cells at each timepoint. **(C)** Percentage of p21-positive cells at the indicated time points after irradiation. **(D)** Log2-transformed average nuclear p21 intensities in p21-positive cells at the indicated time points after irradiation. Error bars in **(C,D)** represent standard error of the mean and standard deviation, respectively. **(E)** Histograms of Log2-transformed average nuclear p21 intensities in p21-positive cells post-irradiation. N = 3 biological replicates for all experiments. *p_*(unadjusted)*_ < 0.05 before Holm-Bonferroni correction.

Using the platform described above, we captured the temporal changes in p21 in individual seeded cells in 3D in response to irradiation. Probability density functions (PDFs) of p21 levels revealed a gradual increase in the proportion of cells expressing high levels of p21 during the first 12 h, with a more apparent increase at 24 h (Figure 2A). These dynamics were also reflected in the cumulative distribution functions (CDFs) of average p21 levels (Figure 2B).

To quantify the fraction of p21-expressing cells and their expression levels, we defined p21-positive cells as those with p21-mCherry intensities in the top 10th percentile at 1 h post-irradiation (Figures 2A,B, dashed lines) and applied this threshold to all subsequent timepoints within the same experiment. Because irradiation occurred before addition of the Matrigel top layer, we could not image the same cells prior to irradiation, making 1 h post-irradiation our earliest available timepoint. Using the top 10th percentile at this timepoint effectively separates the high-intensity tail from the bulk population and minimizes inclusion of background signal. We found that the percentage of p21-positive cells gradually and monotonically increases following irradiation, resulting in approximately 65% p21-positive cells 24 h post-irradiation (Figure 2C). Focusing only on the p21-positive population at each timepoint, we observed a modest increase in p21 intensity between 1 and 4 h after irradiation, which subsequently plateaued over the next hours post-irradiation (Figures 2D,E), suggesting that irradiation of 3D embedded cells leads to a gradual increase in the number of p21-positive cells with the average levels of p21 in those cells remaining relatively constant.

### 3.3 Experimental and computational platforms for visualizing and quantifying spheroids’ responses in 3D

**FIGURE 3:**
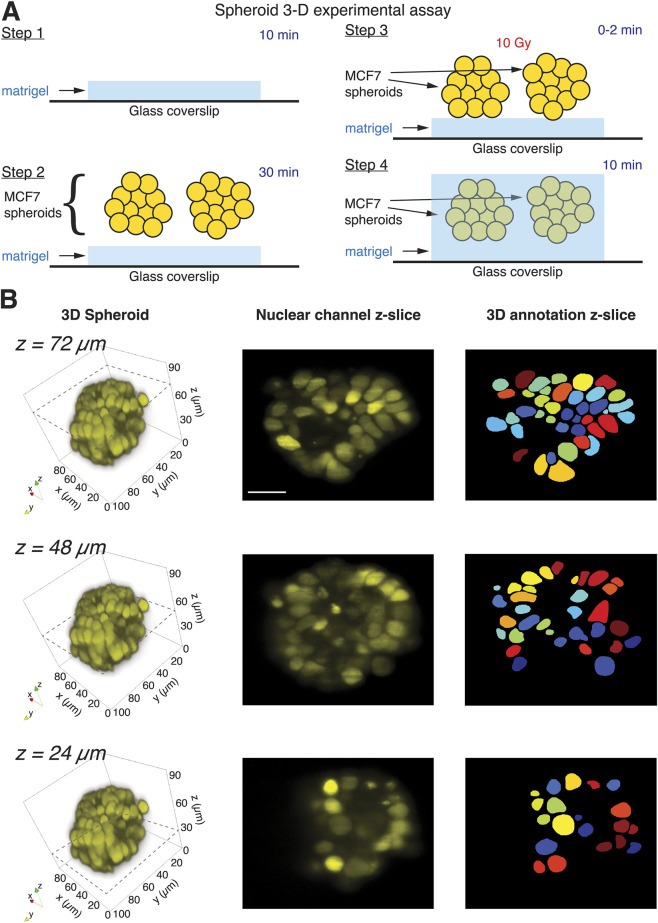
Experimental and analysis pipeline for investigating p21 responses in 3D embedded cancer spheroids. **(A)** Schematic illustration of the experimental assay. Dual-Matrigel layer assay facilitates 3D embedding of irradiated cancer spheroids in planar configuration. **(B)** Light sheet imaging produces high-spatial resolution volumetric stacks of spheroids. Custom MATLAB routines generate VTK files that are exported to ParaView for 3D reconstructions from nuclear channel, with sample z-planes indicated with dashed lines (left column). Corresponding z-slices of microscopy data are shown from z-planes denoted in left column (middle column). Z-planes of 3D annotations for corresponding z-plane, with different colors representing unique computational nuclei identification tags (right column). Scale bar corresponds to 30 μm.

Since protein dynamics in 3D-embedded multicellular structures can be influenced by signaling from both cell-ECM and cell-cell interactions, we next examined the p21 response in spheroids (Paszek et al., 2005; Helmlinger et al., 1997; Kapałczyńska et al., 2016). Our goal was to follow the response of the same spheroid to treatment over time, avoiding the need to average signaling responses from spheroids that may vary in initial size, shape, and cell density. We employed the dual-Matrigel assay described above with spheroids derived from MCF7 cells expressing the MCP-YFP and p21-mCherry reporters (Figure 3A), and imaged irradiated, embedded spheroids using LSFM. Volumetric stacks of spheroids allowed high spatial resolution at multiple depths, revealing areas of varying cell density (Figure 3B, left and middle columns). Note that light sheet imaging occasionally produces faint striping patterns in the nuclear channel, a common artifact in diSPIM systems caused by light sheet scattering and minor refractive index inhomogeneities in 3D-embedded samples (Power et al., 2017). Background subtraction and volumetric averaging in our analysis pipeline ensure that such artifacts do not affect reconstructions, segmentations, or intensity quantifications. We next exported volumetric TIFF files from the nuclear marker channel into the open-source software Napari, which allowed us to assign numeric tags to manually annotated cell nuclei in each slice of our imaging volumes. Using custom MATLAB routines, we linked each 2D nucleus annotation across each slice spanning the nucleus to obtain 3D annotations of cell nuclei, with at least 50 nuclei annotated per spheroid at multiple timepoints post-irradiation (Figure 3B, right column). These annotations were used to quantify p21 levels in individual cells in a spheroid.

### 3.4 Temporal behavior of p21 in irradiated spheroids

**FIGURE 4:**
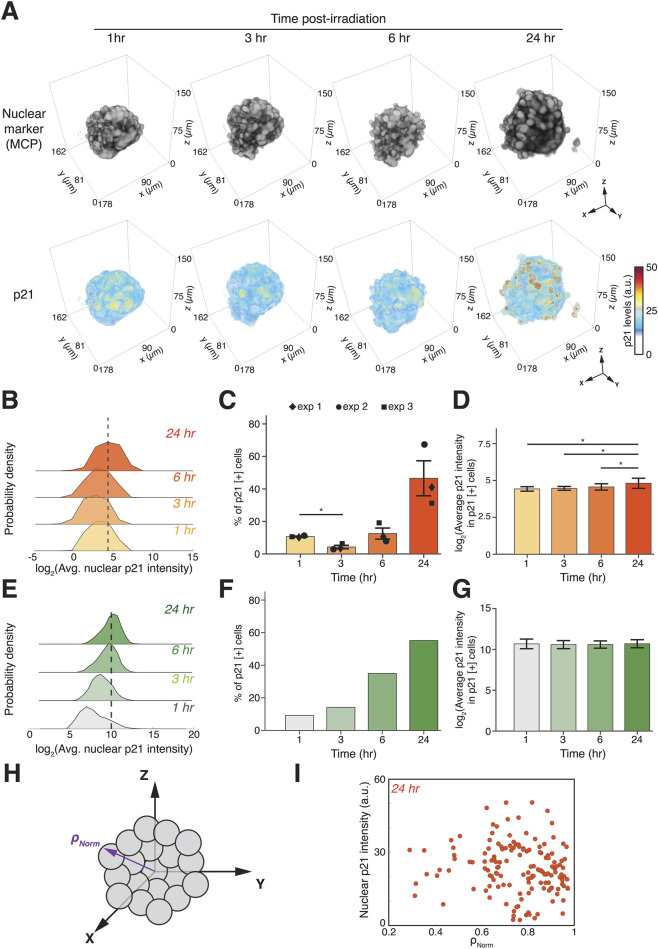
DNA damage increases the fraction of p21-positive cells but not their expression levels, with varying kinetics in spheroids and 2D cultures. **(A)** Representative 3D time-course reconstructions of an irradiated spheroid using nuclear marker (top row) and p21 (bottom row) channels at the indicated timepoints post-irradiation. **(B)** Log2-transformed probability density plots of average nuclear p21 levels in single cells in 10 Gy irradiated spheroids from volumetric light sheet data at the indicated timepoints post-irradiation. Data from one representative spheroid with between 80 and 145 cells annotated and analyzed in each timepoint. **(C)** Percentage of p21-positive cells within irradiated spheroids at the indicated timepoints post-irradiation. Data averaged from three independently irradiated spheroids. **(D)** Log2-transformed average p21 levels in p21-positive cells within irradiated spheroids. **(E–G)** Log2-transformed probability density plots of average nuclear p21 levels **(E)**, percentage of p21-positive cells **(F)** and Log2-transformed average p21 levels in p21-positive cells **(G)** in 10 Gy irradiated 2D cultured cells at the indicated timepoints post-irradiation. Error bars in **(C)** represent standard error of the mean. Error bars in **(D,F)** represent standard deviation. Data in **(E–G)** taken from Hafner et al. (2020). **(H)** Schematic illustrating the normalized coordinate system for defining cell positions as a function of distance between spheroid centroids to edges *ρ*_*Norm*_. **(I)** Scatter plots of average nuclear p21 intensities in cells as a function of *ρ*_*Norm*_ for single cells in irradiated spheroid 24 h post-irradiation. *p_*unadjusted*_ < 0.05 before Holm-Bonferroni correction.

To quantify the response of p21 to irradiation in spheroids we first reconstruct 3D multicellular structures based on the MCP-YFP nuclear marker (Figure 4A, top row). We noted that spheroids remained relatively intact during the 24 h imaging period with only modest spreading at 24 h (Figure 4A, top row). Heatmaps of p21-mCherry levels in the reconstructed spheroids showed only a mild increase in p21 levels during the first 6 h after irradiation, with a stronger induction at the 24 h timepoint (Figure 4A, bottom row). To quantify the fraction of p21-positive cells, we again defined a threshold using 10% at 1 h post-irradiation as the baseline, allowing us to compare relative changes within the same spheroid and compare the results with those obtained using individually dispersed cells (Figure 2). We found that the percentage of p21-positive cells decreased at 3 h post-irradiation, followed by a modest increase at 6 h and a more evident increase at 24 h, reaching almost 50% p21-positive cells (Figures 4B,C). The levels of p21 in p21-positive cells within spheroids remained relatively constant from 1–6 h post-irradiation and only mildly but significantly increased at 24 h (Figure 4D).

We next compared p21 responses in irradiated cells in 3D to those of irradiated cells in 2D cultures in the absence of ECM (Hafner et al., 2020). To maintain consistency between the 3D and 2D analyses, we used the 1 h post-irradiation time point of cells grown in 2D as the baseline for these experiments. Probability densities of p21 levels in irradiated cells cultured in 2D revealed an increase in p21 levels over time, with the highest levels observed 24 h post-irradiation (Figure 4E). The percentage of p21-positive cells in 2D increased monotonically (Figure 4F), resembling the response of individually dispersed cells in 3D (Figure 2C), but different from the response in spheroids (Figure 4C). The average p21 levels in p21-positive cells remained constant, with large heterogeneity in p21 expression levels as indicated by the large standard deviations (Figure 4G). It is important to note that while the comparison of our newly collected data in 3D with the 2D data collected by us in Hafner et al., 2020 is based on the same clonal line and standardized conditions, it remains an indirect comparison and should be interpreted with this limitation in mind.

Lastly, we expanded our computational analysis to determine the spatial dependency of p21 induction in spheroids. We annotated cells within spheroids as a function of their distance from geometric spheroid centroids *ρ* (Figure 4H) and normalized the radial distances by defining the distance from centroids to spheroid edges as ${0\leq \rho }_{Norm}\leq 1$ for each cell. The relationship between average p21 levels and *ρ*_*Norm*_ 24 h after irradiation showed no dependency between cells’ spatial positions within the spheroids and their p21 levels (Figure 4I). Note that due to the higher density of cells at centroids, our method is limited in its ability to clearly annotate and analyze cells close to this position. Similar tools can be used to assess whether the levels and dynamics of other signaling molecules depend on cells’ spatial position within spheroids.

## 4 Discussion

We have developed experimental assays and computational tools to investigate the dynamics of signaling pathways in 3D environments. We focused on the response of the cell cycle regulator p21 to irradiation and compared its behavior in individually dispersed cells and in spheroids with our previous data on p21 responses in traditional 2D culture cells. In all systems, we used a single 10 Gy dose of ionizing radiation, a dose within the range used in certain stereotactic or ablative regimens and a standard single-fraction dose in many preclinical mechanistic studies. Overall, our work revealed that some aspects of the p21 response to irradiation in spheroids are comparable to its response in 2D culture. First, both spheroids and 2D cultures showed approximately 50% p21-positive cells at 24 h post-irradiation. Previous work showed that p21 induction in response to irradiation in 2D depends on the cell cycle, with no induction in cells irradiated in S phase (Hafner et al., 2020; Stewart-Orns et al., 2016). Similar cell-cycle dependency may exist in the regulation of p21 in irradiated spheroids. In addition, the magnitude of p21 levels in p21-positive cells remained relatively constant or was only mildly increased across all experimental systems (2D, 3D dispersed cells, and 3D spheroids). This finding aligns with studies showing that p53, the transcription factor activating p21, oscillates in response to irradiation and, each pulse regulates the probability, rather than the magnitude, of p21 transcription (Hafner et al., 2020). A closer look at the dynamics of p21 induction revealed a few quantitative differences between 2D and spheroids. First, in 2D culture, as well as 3D culture of individually dispersed cells, the fraction of p21-positive cells increased gradually at a constant rate (Figures 2C, 4F), while in spheroids, the fraction of positive cells decreased at 3 h, remained relatively low at 6 h and increased significantly only at 24 h (Figure 4C). The decrease in p21-positive cells at 3 h was captured across multiple spheroids, suggesting that it represents real behavior rather than noise or an imaging artifact. The cause of such a decrease is unknown. One plausible explanation could be that cells with high p21 levels may be more prone to early apoptosis or mitotic catastrophe after irradiation. Alternatively, p21 degradation may be enhanced in 3D spheroids, while transcriptional upregulation by p53 has not yet fully kicked in, leading to a transient net decrease. Further studies involving fluorescent reporters for known p21 regulators, such as p53, coupled with high-content imaging platforms that allow following the same single cell within a spheroid would help reveal the molecular mechanism leading to the transient decrease in p21-positive cells post-irradiation.

An additional factor that may contribute to slower p21 induction in spheroids is the potential attenuation of ionizing radiation. At the same dose, outer cell layers may partially shield inner cells, effectively reducing the dose, and hence DNA damage, experienced by the spheroid core compared to dispersed 2D cultures. Even though our analysis revealed no dependency between cells’ spatial positions within the spheroids and their p21 levels, future work combining dose-response measurements with spatial readouts of DNA damage (γH2AX staining or live reporters) will be important to more directly assess the potential shielding effect. More broadly, although these 3D spheroid assays provide mechanistic insight into DNA damage responses in a context that is more complex than 2D culture, we recognize that human tumors differ substantially from spheroids in their size and geometry, microenvironmental heterogeneity, vascularization, stromal and immune components, and overall treatment context. Thus, our results should be interpreted as a controlled framework for understanding p21 regulation in 3D, rather than as a direct surrogate for clinical tumor behavior or outcomes in patients receiving radiotherapy.

Our pipeline provides methods to measure and compare single-cell responses in 3D systems, which is critical for capturing cellular heterogeneities in signaling responses. Previous work using a live-cell reporter to measure p21 in response to the DNA damaging-drug Etoposide, revealed dose-dependent induction 24 h after drug treatment by averaging spheroid cross-sections (Mondesert et al., 2015). While this work was the first to use live-cell imaging to quantify p21 responses to DNA damage, bulk p21 measurements did not allow for assessing cellular heterogeneities and comparisons between corresponding 2D studies where mechanisms of p21 and other elements of the DNA damage response have been well-defined. Given that other key components of the DNA damage response, such as p53, have been shown to exhibit cell-to-cell heterogeneity in cell culture (Peak et al., 2016; Reyes et al., 2018) and *in vivo* (Simerzin et al., 2025), future studies using the approaches outlined in the present study may be useful in further interrogating the DNA damage response in 3D multicellular structures at the single-cell level.

An important open question arising from our work concerns the downstream functional consequences of p21 dynamics for cellular behavior, including effects on cell-cycle phase distributions, cell motility, and cell morphology. Future work combining long-term single-cell tracking with cell-cycle reporters and biophysical measurements will be required to link p21 dynamics more directly to changes in cell proliferation, migration, and tumorigenic potential. In this regard, recent work under 3D hypoxic conditions has begun to dissect how microenvironmental stress and p53-pathway activity shape early tumorigenesis through coupled genetic and mechanical instabilities, providing a useful conceptual framework for extending our analysis of irradiation-induced p21 dynamics to broader tumorigenic mechanisms (Wang et al., 2025a).

While our experimental assay and computational analysis facilitate studying the response of irradiated spheroids, the need for manual annotations in our pipeline remains a bottleneck in performing high-throughput studies of signaling responses in 3D at the single-cell level. The development of novel automated cell segmentation algorithms using approaches such as convolutional neural network classifiers for spheroids, which have been applied in other systems (Eismann et al., 2020; Weigert et al., 2020; Stringer et al., 2021), may address this limitation in the future. Automated nuclei segmentation would also facilitate the study of these signaling responses with higher temporal resolution and for longer periods of time, providing a more detailed description of signaling dynamics in 3D systems.

## Data Availability

The raw data supporting the conclusions of this article will be made available by the authors, without undue reservation.

## References

[B1] Abbas T. Dutta A. (2009). p21 in cancer: intricate networks and multiple activities. Nat. Rev. Cancer 9 (6), 400–414. 10.1038/nrc2657 19440234 PMC2722839

[B2] Balcer-Kubiczek E. K. Yin J. Lin K. Harrison G. H. Abraham J. M. Meltzer S. J. (1995). p53 mutational status and survival of human breast cancer MCF-7 cell variants after exposure to X rays or fission neutrons. Radiat. Research 142 (3), 256–262. 10.2307/3579133 7761574

[B3] Bhuker S. Sinha A. K. Arora A. Tuli H. S. Datta S. Saini A. K. (2025). Genes and proteins expression profile of 2D vs 3D cancer models: a comparative analysis for better tumor insights. Cytotechnology 77 (2), 51. 10.1007/s10616-025-00714-w 39867829 PMC11759753

[B4] Breslin S. O’Driscoll L. (2016). The relevance of using 3D cell cultures, in addition to 2D monolayer cultures, when evaluating breast cancer drug sensitivity and resistance. Oncotarget 7 (29), 45745–45756. 10.18632/oncotarget.9935 27304190 PMC5216757

[B5] Cambra H. M. Tallapragada N. P. Mannam P. Breault D. T. Klein A. M. (2022). Triple‐decker sandwich cultures of intestinal organoids for long‐term live imaging, uniform perturbation, and statistical sampling. Curr. Protoc. 2 (1), e330. 10.1002/cpz1.330 35030297 PMC9006308

[B6] Chow D. J. Schartner E. P. Corsetti S. Upadhya A. Morizet J. Gunn-Moore F. J. (2024). Quantifying DNA damage following light sheet and confocal imaging of the Mammalian embryo. Sci. Rep. 14 (1), 20760. 10.1038/s41598-024-71443-x 39237572 PMC11377761

[B7] Doublier S. Belisario D. C. Polimeni M. Annaratone L. Riganti C. Allia E. (2012). HIF-1 activation induces doxorubicin resistance in MCF7 3-D spheroids *via* P-glycoprotein expression: a potential model of the chemo-resistance of invasive micropapillary carcinoma of the breast. BMC Cancer 12 (1), 4. 10.1186/1471-2407-12-4 22217342 PMC3262753

[B8] Eismann B. Krieger T. G. Beneke J. Bulkescher R. Adam L. Erfle H. (2020). Automated 3D light-sheet screening with high spatiotemporal resolution reveals mitotic phenotypes. J. Cell Sci. 133 (11), jcs245043. 10.1242/jcs.245043 32295847 PMC7286290

[B9] El-Deiry W. S. Tokino T. Velculescu V. E. Levy D. B. Parsons R. Trent J. M. (1993). WAF1, a potential mediator of p53 tumor suppression. Cell 75 (4), 817–825. 10.1016/0092-8674(93)90500-p 8242752

[B10] Frantz D. Karamahmutoglu T. Schaser A. J. Kirik D. Berrocal E. (2022). High contrast, isotropic, and uniform 3D-imaging of centimeter-scale scattering samples using structured illumination light-sheet microscopy with axial sweeping. Biomed. Opt. Express 13 (9), 4907–4925. 10.1364/BOE.464039 36187271 PMC9484431

[B11] Hafner A. Reyes J. Stewart-Ornstein J. Tsabar M. Jambhekar A. Lahav G. (2020). Quantifying the central dogma in the p53 pathway in live single cells. Cell Systems 10 (6), 495–505. 10.1016/j.cels.2020.05.001 32533938 PMC7413213

[B12] Helmlinger G. Netti P. A. Lichtenbeld H. C. Melder R. J. Jain R. K. (1997). Solid stress inhibits the growth of multicellular tumor spheroids. Nat. Biotechnol. 15 (8), 778–783. 10.1038/nbt0897-778 9255794

[B13] Imamura Y. Mukohara T. Shimono Y. Funakoshi Y. Chayahara N. Toyoda M. (2015). Comparison of 2D- and 3D-culture models as drug-testing platforms in breast cancer. Oncol. Rep. 33 (4), 1837–1843. 10.3892/or.2015.3767 25634491

[B14] Kapałczyńska M. Kolenda T. Przybyła W. Zajączkowska M. Teresiak A. Filas V. (2016). 2D and 3D cell cultures – a comparison of different types of cancer cell cultures. Archives Med. Sci. 14 (4), 910–919. 10.5114/aoms.2016.63743 30002710 PMC6040128

[B15] Mondesert O. Frongia C. Clayton O. Boizeau M.-L. Lobjois V. Ducommun B. (2015). Monitoring the activation of the DNA damage response pathway in a 3D spheroid model. PloS One 10 (7), e0134411. 10.1371/journal.pone.0134411 26225756 PMC4520595

[B16] Moysidou C.-M. Barberio C. Owens R. M. (2021). Advances in engineering human tissue models. Front. Bioeng. Biotechnol. 8, 620962. 10.3389/fbioe.2020.620962 33585419 PMC7877542

[B17] Nürnberg E. Vitacolonna M. Klicks J. Von Molitor E. Cesetti T. Keller F. (2020). Routine optical clearing of 3D-Cell cultures: simplicity forward. Front. Mol. Biosci. 7, 20. 10.3389/fmolb.2020.00020 32154265 PMC7046628

[B18] Paszek M. J. Zahir N. Johnson K. R. Lakins J. N. Rozenberg G. I. Gefen A. (2005). Tensional homeostasis and the malignant phenotype. Cancer Cell 8 (3), 241–254. 10.1016/j.ccr.2005.08.010 16169468

[B19] Peak A. Liu J. Loewer A. Forrester W. Lahav G. (2016). Cell-to-Cell variation in p53 dynamics leads to fractional killing. Cell 165 (3), 631–642. 10.1016/j.cell.2016.03.025 27062928 PMC5217463

[B20] Power R. M. Huisken J. (2017). A guide to light-sheet fluorescence microscopy for multiscale imaging. Nat. Methods 14 (4), 360–373. 10.1038/nmeth.4224 28362435

[B21] Reyes J. Chen J.-Y. Stewart-Ornstein J. Karhohs K. W. Mock C. S. Lahav G. (2018). Fluctuations in p53 signaling allow escape from cell-cycle arrest. Mol. Cell 71 (4), 581–591.e585. 10.1016/j.molcel.2018.06.031 30057196 PMC6282757

[B22] Richardson D. S. Lichtman J. W. (2015). Clarifying tissue clearing. Cell 162 (2), 246–257. 10.1016/j.cell.2015.06.067 26186186 PMC4537058

[B23] Simerzin A. Ackerman E. E. Fujimaki K. Kohler R. H. Iwamoto Y. Heltberg M. S. (2025). Cell confluency affects p53 dynamics in response to DNA damage. Mol. Biol. Cell 36 (6), br16. 10.1091/mbc.E24-09-0394 40202833 PMC12206509

[B24] Stewart-Ornstein J. Lahav G. (2016). Dynamics of CDKN1A in single cells defined by an endogenous fluorescent tagging toolkit. Cell Rep. 14 (7), 1800–1811. 10.1016/j.celrep.2016.01.045 26876176 PMC5154611

[B25] Stringer C. Wang T. Michaelos M. Pachitariu M. (2021). Cellpose: a generalist algorithm for cellular segmentation. Nat. Methods 18 (1), 100–106. 10.1038/s41592-020-01018-x 33318659

[B26] Tallapragada N. P. Cambra H. M. Wald T. Jalbert S. K. Abraham D. M. Klein O. D. (2021). Inflation-collapse dynamics drive patterning and morphogenesis in intestinal organoids. Cell Stem Cell 28 (9), 1516–1532. 10.1016/j.stem.2021.04.002 33915079 PMC8419000

[B27] Wang Z. Tian L. Li B. (2025a). Hypoxia-induced active dynamics promotes early tumorigenesis. bioRxiv., 630973. 10.1101/2024.12.31.630973

[B28] Wang Z. Tian L. Li B. (2025b). Neurogenesis leads early development in zebrafish. bioRxiv., 687769. 10.1101/2025.11.12.687769

[B29] Weigelt B. Lo A. T. Park C. C. Gray J. W. Bissell M. J. (2010). HER2 signaling pathway activation and response of breast cancer cells to HER2-targeting agents is dependent strongly on the 3D microenvironment. Breast Cancer Res. Treat. 122 (1), 35–43. 10.1007/s10549-009-0502-2 19701706 PMC2935800

[B30] Weigert M. Schmidt U. Haase R. Sugawara K. Myers G. (2020). “Star-convex polyhedra for 3D object detection and segmentation in microscopy,” in Paper presented at: proceedings of the IEEE/CVF winter conference on applications of computer vision.

[B31] Xie R. Pal V. Yu Y. Lu X. Gao M. Liang S. (2024). A comprehensive review on 3D tissue models: biofabrication technologies and preclinical applications. Biomaterials 304, 122408. 10.1016/j.biomaterials.2023.122408 38041911 PMC10843844

[B32] Zhang G. Liang Q. Wu Y. Wang Y. (2025). Insights on the differences between two-and three-dimensional culture systems in tumor models. Int. J. Mol. Med. 56 (5), 1–16. 10.3892/ijmm.2025.5626 PMC12425351 40910266

